# In vivo imaging of the hyaloid vascular regression and retinal and choroidal vascular development in rat eyes using optical coherence tomography angiography

**DOI:** 10.1038/s41598-020-69765-7

**Published:** 2020-07-30

**Authors:** Yongjoo Kim, Jang Ryul Park, Hye Kyoung Hong, Myounghee Han, Jingu Lee, Pilhan Kim, Se Joon Woo, Kyu Hyung Park, Wang-Yuhl Oh

**Affiliations:** 1 Department of Mechanical Enginnereing, Korea Advanced Institute of Science and Technology (KAIST), 291 Daehak-ro, Yuseong-gu, Daejeon, 34141 Republic of Korea; 2 KI for Health Science and Technology, Korea Advanced Institute of Science and Technology (KAIST), 291 Daehak-ro, Yuseong-gu, Daejeon, 34141 Republic of Korea; 3 Department of Ophthalmology, Seoul National University College of Medicine, Seoul National University Bundang Hospital (SNUBH), 173-82 Gumi-ro, Bundang-gu, Seongnam, Gyeongi-do 13620 Republic of Korea; 4 Machine Vision R&D Center, Koh Young Technology, Inc., Yongin-si, Gyeonggi-do 16864 Republic of Korea; 5 Graduate School of Nanoscience and Technology, Korea Advanced Institute of Science and Technology (KAIST), 291 Daehak-ro, Yuseong-gu, Daejeon, 34141 Republic of Korea

**Keywords:** Optical imaging, Angiogenesis, Biomedical engineering

## Abstract

This study investigates the hyaloid vascular regression and its relationship to the retinal and choroidal vascular developments using optical coherence tomography angiography (OCTA). Normal and oxygen-induced retinopathy (OIR) rat eyes at postnatal day 15, 18, 21, and 24 were longitudinally imaged using OCTA. At each day, two consecutive imaging for visualizing the hyaloid vasculature and the retinal and choroidal vasculatures were conducted. The hyaloid vessel volume and the retinal and choroidal vessel densities were measured. The hyaloid vessel volumes gradually decreased during the regression, although the OIR eyes exhibited large vessel volumes at all time points. A spatial relationship between persistent hyaloid vasculature and retardation of underlying retinal vascular development was observed in the OIR eyes. Furthermore, anti-vascular endothelial growth factor (VEGF) was administered intravitreally to additional OIR eyes to observe its effect on the vascular regression and development. The VEGF injection to OIR eyes showed reduced persistent hyaloid vessels in the injected eyes as well as in the non-injected fellow eyes. This study presents longitudinal imaging of intraocular vasculatures in the developing eye and shows the utility of OCTA that can be widely used in studies of vascular development and regression and preclinical evaluation of new anti-angiogenic drugs.

## Introduction

Hyaloid vasculature is one of the major blood supply sources in the developing eye and plays an important role in the growth and maturation of crystalline lens, vitreous, and retina^1^. It consists of a group of vascular components: hyaloid artery, vasa hyaloidea propria (VHP), and tunica vasculosa lentis (TVL). The hyaloid artery at the optic nerve perfuses intraocular blood flow and ramifies across the vitreous space through VHP, which anastomoses with TVL forming a capillary network that surrounds the posterior surface of the crystalline lens. At the margin of the optic cup, the TVL anastomoses with the annular vessels and is connected to the choroidal vasculature^2^. In the late stages of ocular vessel development, the hyaloid vasculature spontaneously regresses and completely disappears around mid-gestation in humans and around birth in rodents^2,3^, by which time the retina is fully vascularized. In the human eye, failure of hyaloid regression and persistent hyaloid vessels referred to as persistent hyper plastic primary vitreous can result in retinal hemorrhage and detachment^4^. Therefore, it is important to understand the mechanisms and key factors that regulate vascular formation and regression during development.

The hyaloid regression is initiated with the apoptosis of vascular endothelial cells and is associated with intraocular macrophages^5,6^, vascular endothelial growth factor (VEGF) level^7,8^, and reduced hemodynamic force exerting on the endothelial cells^9^. To expand the understanding of the hyaloid regression, numerous studies have tried to reveal specific signal pathways and the roles of related genes or molecules^10–14^. Furthermore, since the hyaloid vasculature is known to be functionally and anatomically linked to retinal and choroidal vasculatures^2,15,16^, a comprehensive understanding of the hyaloid regression and its relationship with the retinal and choroidal vascular development is highly desired, thereby increasing the importance of establishing imaging techniques for concurrently visualizing different vasculatures in the developing eye.

There have been a number of imaging techniques for visualizing hyaloid vasculature. Studies using the scanning electron microscopy observed the hyaloid vasculatures of the human fetuses, pigs, and mice in fine detail^17–19^. The whole mount microscopy is also utilized for visualizing the entire hyaloid vasculature. These techniques, however, inherently require ex vivo tissues for imaging and cannot capture the dynamic changes of the vessels in individual eyes. An in vivo study using scanning laser ophthalmoscope (SLO) observed hyaloid vasculature in a mouse eye, showing the vascular regression at multiple time points^20^.

Optical coherence tomography angiography (OCTA) is a recently developed imaging technique that visualizes vascular networks in vivo. One of the main advantages of OCTA is its capability of depth-resolved imaging and assessing three-dimensional vascular morphology. The application of OCTA to clinical ophthalmic imaging has shown its utility in diagnosing vascular abnormalities in the anterior segment^21^ and retinal^22,23^ and choroidal layers^24,25^. There have also been imaging studies on various animal disease models such as laser-induced choroidal neovascularization^26,27^, oxygen-induced retinopathy^28^, elevated intraocular pressure^29^, and targeted retinal vessel occlusion using OCTA^30^.

In this study, we present longitudinal OCTA imaging of hyaloid vasculature in neonatal rat eyes in vivo. In addition to normal rats, a rat model of oxygen-induced retinopathy (OIR) was used to induce persistent hyaloid vessels and to compare the regression in normal and pathologic conditions. Right after hyaloid imaging, the retinal and choroidal vasculatures were also imaged in the same eyes to investigate relationships between the hyaloid regression and the retinal and choroidal vascular developments. In addition, an anti-VEGF agent was injected to the OIR eyes and its effect on the hyaloid regression as well as the retinal and choroidal vascular development was investigated.

## Results

### Visualization of hyaloid vasculature using OCTA

**Figure 1:**
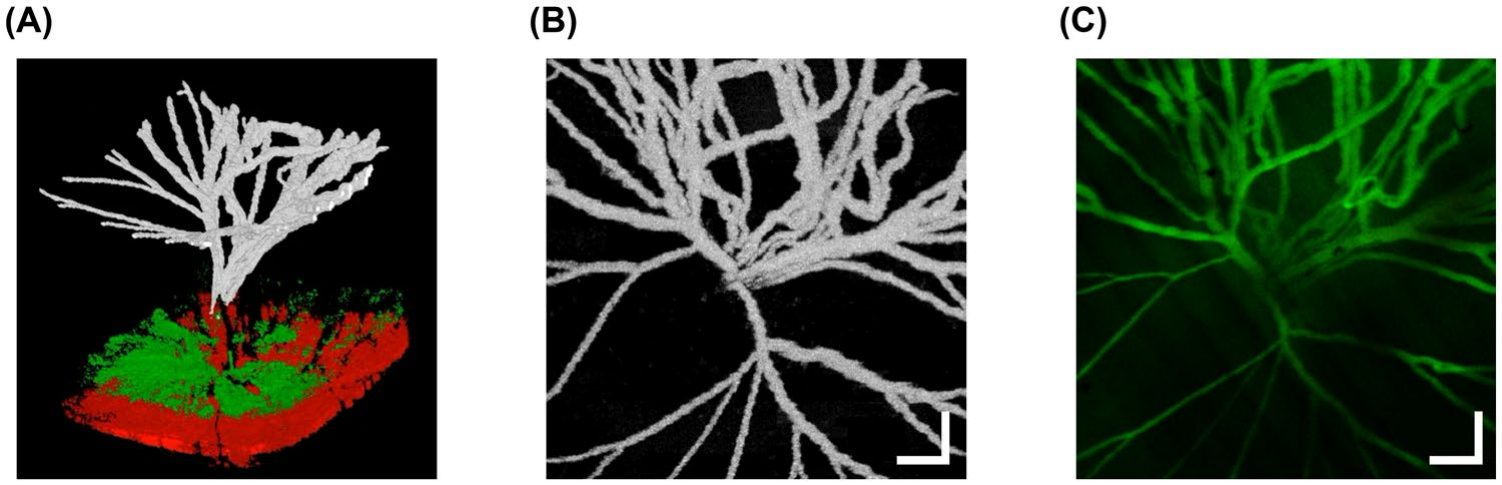
Visualization of hyaloid vasculature of an OIR eye at P24. (**a**) A three-dimensional view of the posterior part of the eye. The hyaloid vessels, the retinal and choroidal tissues are represented as white, green, and red colors, respectively. (**b**) An en face projection OCTA image of the hyaloid vasculature. (**c**) An FA image of the hyaloid vasculature in the same eye. Scale bars: 200 μm.

Figure 1 shows images of the hyaloid vasculature observed in an OIR eye at P24. Figure 1a represents a three-dimensional view of the posterior part of the eye consisting of the hyaloid vessels reconstructed from the hyaloid angiogram, and retinal and choroidal tissues reconstructed from the structural OCT data, each of which is color-coded as white, green, and red, respectively (Supplementary Video S1). The hyaloid angiogram is segmented and projected to generate an en face hyaloid OCTA image as shown in Fig. 1b. Figure 1c is the FA image acquired from the same eye. The vascular morphology in the FA image shows good agreement with that in the OCTA image.

### Temporal changes of the hyaloid vasculature

**Figure 2:**
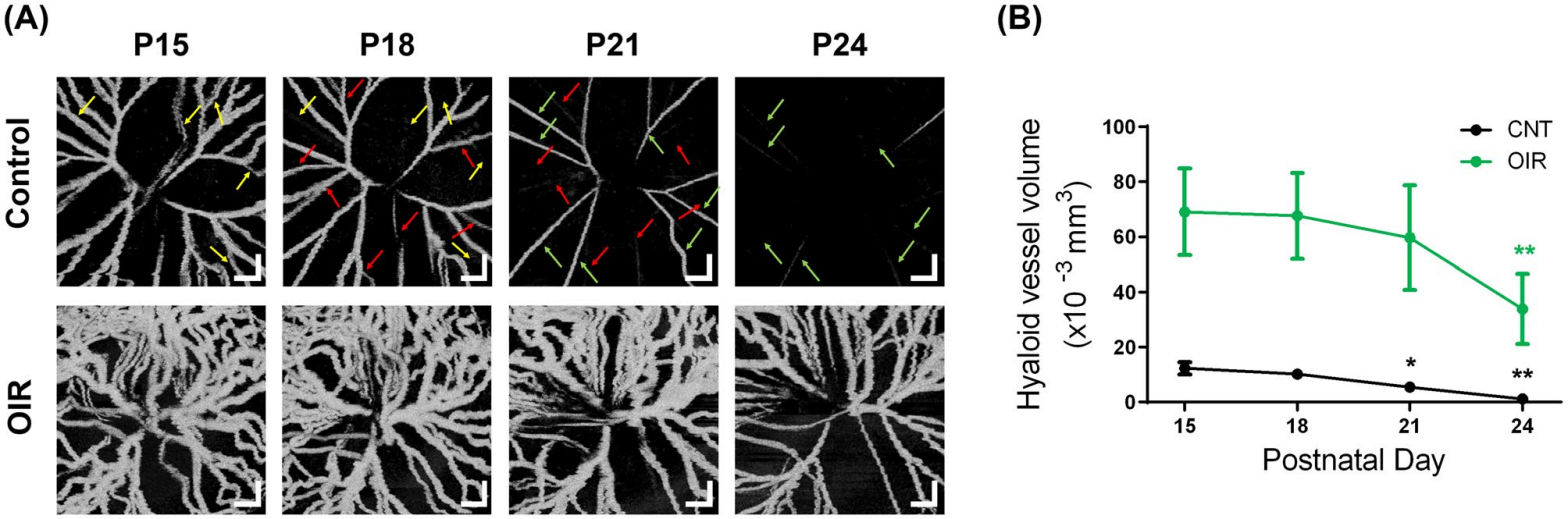
Longitudinal observation of the hyaloid regression from P15 to P24. (**a**) En face hyaloid OCTA images in control (upper row) and OIR eyes (lower row). The yellow, red, and green arrows in the OCTA images of the control eye indicate the vessels that were present at P15, P18, and P21 but disappeared at P18, P21, and P24, respectively. Scale bars: 200 μm. (**b**) Hyaloid vessel volume measurement in the control and the OIR eyes. *P < 0.05 and **P < 0.01.

Figure 2a shows the longitudinal changes of the hyaloid vasculatures in control and OIR eyes from P15 to P24. In both control and OIR eyes, the hyaloid vasculatures gradually regressed over time. In the control eye, it is notable that the regression did not take place simultaneously in the entire vessels. For example, the vessels indicated by the yellow arrows at P15 were profoundly missing at P18. In the same manner, the vessels indicated by the red and green arrows at P18 and P21 disappeared at P21 and P24, respectively. This observation is consistent with the result in the previous study showing segmental regression of the hyaloid vessel^1^. Compared to the normal eye, the OIR eye exhibited a persistence of the hyaloid vessels and the vessels are significantly dilated and tortuous at all time points because of the exposure to hyperoxia, which induces inhibited hyaloid regression and abnormal vascular phenotypes^31^.

For quantitative analysis of the regression, the hyaloid vessel volumes are measured as shown in Fig. 2b. The vessel volumes in control eyes decreased at a nearly constant rate. The OIR eyes exhibited larger vessel volumes compared to control eyes due to oxygen exposure. The vessel volume in the OIR eyes showed no significant decrease from P15 to 21 but a decrement at P24.

### Spatial correlation between the hyaloid vascular regression and retinal and choroidal vascular developments

**Figure 3:**
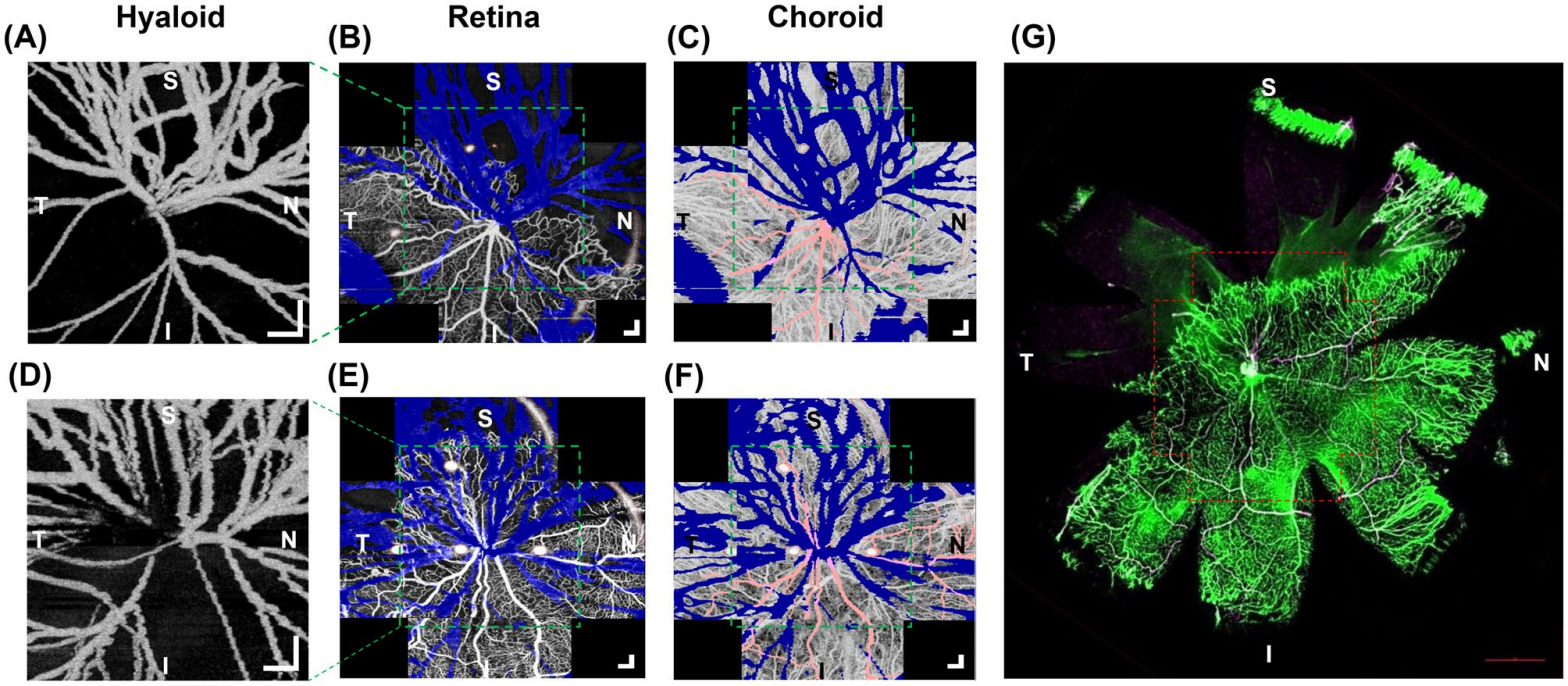
Visualization of the hyaloid, retinal, and choroidal vasculatures of the OIR eyes at P24. (**a**) Hyaloid OCTA image of an OIR eye (same eye shown in Fig. 1). (**b**,**c**) Retinal and choroidal OCTA images of the same eye. The green dotted boxes indicate the regions that correspond to the imaging field of the hyaloid OCTA. S, N, I, and T on the images represent the superior, nasal, inferior, and temporal part of the eye, respectively. Artifacts enclosed by the red-dotted contours are caused by spurious reflections from the imaging optics of the OCTA system. (**d**) Hyaloid OCTA image of another OIR eye (same eye shown in Fig. 2). (**e**,**f**) Retinal and choroidal OCTA images of the corresponding eye. Scale bars: 200 μm. (**g**) Retinal whole mount image of the same eye. The red dotted contour matches to the imaging field of view of the retinal OCTA image. Scale bars: 1 mm.

Figure 3 shows OCTA images of the hyaloid, retinal, and choroidal vasculatures acquired from two OIR eyes at P24. For the retina and choroid OCTA, five images were acquired from different locations by rotating the pup’s body to produce a wide-field composite image. The blue areas in the retina and choroid OCTA images indicate local regions with low signal due to the attenuation of the OCT beam by the hyaloid vessels. Figure 3a shows that the hyaloid vessels persisted predominantly in the superior and nasal directions whereas the vessels in inferior and temporal directions regressed substantially. This spatial vessel distribution was correspondingly observed in the retinal OCTA of the same eye showing that the retinal vascular development was retarded in the superior and nasal directions as shown in Fig. 3b. In contrast, the choroidal vasculature in Fig. 3c did not show a noticeable relevant spatial vessel distribution. Similarly, in Fig. 3d–f, OCTA images acquired from another OIR eye showed persistent hyaloid vessels in the superior and temporal directions where the retinal vasculature was retarded. Again, the choroidal vasculature did not show any spatially varying vascular retardation. (OCTA images from additional control and OIR eyes are provided in Supplementary Fig. 1) The retinal whole mount image of the same eye (Fig. 3g) shows a good match to the retinal OCTA shown in Fig. 3e. The red-dotted box in the retinal whole mount image corresponds to the imaging field of view of the OCTA. A wider avascular region observed in Fig. 3g confirms the retinal vascular retardation in the superior and temporal directions as observed in Fig. 3e.

**Figure 4:**
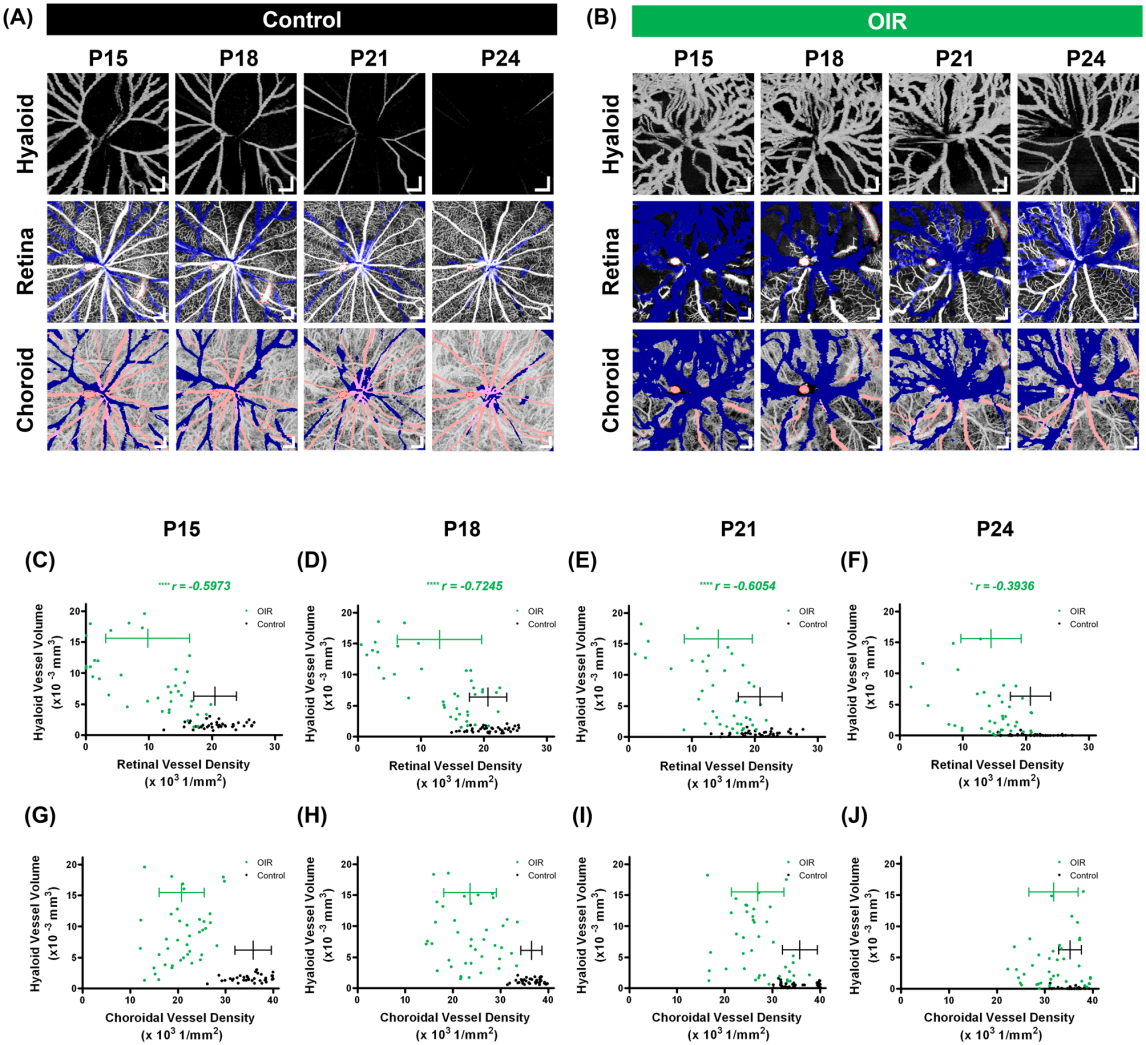
Longitudinal observation of the hyaloid, retinal, and choroidal vasculatures in the control and the OIR eyes. (**a**,**b**) En face OCTA images of the three vasculatures in the control and OIR eyes (same eyes shown in Fig. 2), respectively. Artifacts enclosed by the red-dotted contours are caused by spurious reflections from the imaging optics of the OCTA system. Scale bars: 200 μm. (**c**–**f**) Correlation analyses between the hyaloid vessel volumes and the retinal vessel densities in the control (black) and the OIR (green) eyes. *P < 0.05 and ****P < 0.0001. (**g**–**j**) Correlation analyses between the hyaloid vessel volumes and the choroidal vessel densities in the control (black) and the OIR (green) eyes.

Figure 4a,b show the hyaloid, retinal, and choroidal vasculatures observed in control and OIR eyes (same eyes shown in Fig. 2), respectively, from P15 to P24. To analyze the aforementioned relationship of spatial vessel distribution between three vasculatures quantitatively, hyaloid vessel volumes and retinal and choroidal vessel densities were measured as shown in Fig. 4c–j. A total of 40 data points [5 enrolled eyes and 8 subsets per OCTA image (Supplementary Fig. 2)] were plotted for control and OIR eyes at each time point. Figure 4c–f show correlations between the hyaloid vessel volume and the retinal vessel density. In OIR eyes, the hyaloid vessel volumes have negative correlations with the retinal vessel densities at all time points. This implies that there exists a spatial correlation between the hyaloid regression and the retinal vascular development. At P24, the correlation was not as high as those measured in P15 to P21 and we attribute this to the spontaneous retinal vascularization, which is known as general pathophysiology in OIR models^32^. The control eyes did not show any correlation between the two variables. In contrast, the choroidal vessel densities in Fig. 4g–j did not have any spatial correlation with the hyaloid vessel volumes in either group.

### Effect of intravitreal anti-VEGF injection on the regression of hyaloid vasculature

**Figure 5:**
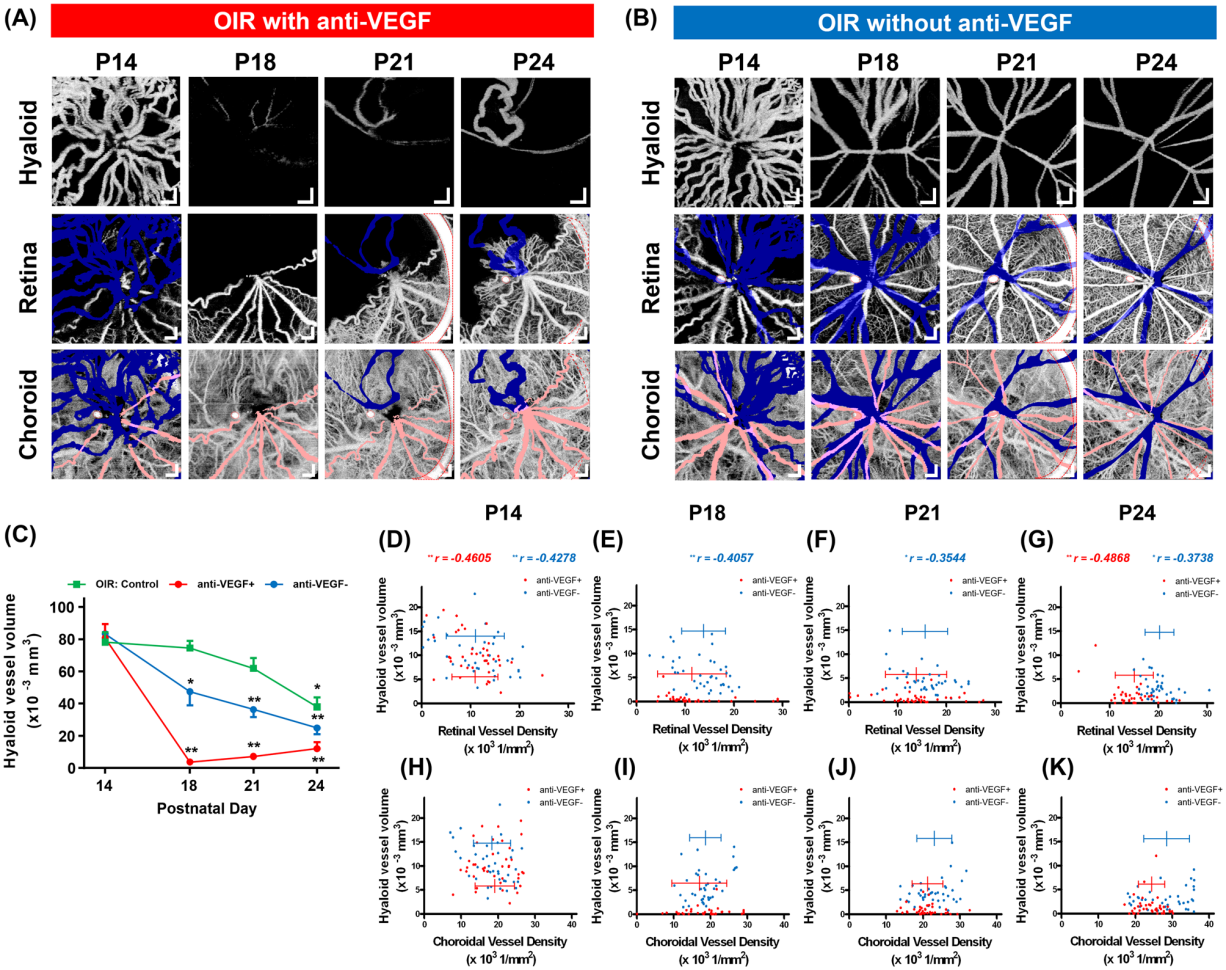
Longitudinal observation of the hyaloid, retinal, and choroidal vasculatures in the OIR eyes that received an anti-VEGF injection and the fellow eyes. (**a**,**b**) En face OCTA images of the three vasculatures in the injected and the fellow eyes, respectively. Artifacts enclosed by the red-dotted contours are caused by spurious reflections from the imaging optics of the OCTA system. Scale bars: 200 μm. (**c**) Hyaloid vessel volume measurement in the OIR eyes that have never received an anti-VEGF injection (green line), the OIR eyes with the injection (red line), and their fellow eyes (blue line). *P < 0.05 and **P < 0.01. (**d**–**g**) Correlation analyses between the hyaloid vessel volumes and the retinal vessel densities in the injected (red) and the fellow (blue) eyes. *P < 0.05 and **P < 0.01. (**h**–**k**) Correlation analyses between the hyaloid vessel volumes and the choroidal vessel densities in the injected (red) and the fellow (blue) eyes.

Figure 5a,b show the hyaloid, retinal, and choroidal vasculatures in the OIR eye that received an anti-VEGF (aflibercept) intravitreal injection and their non-injected fellow eye, respectively, from P14 to P24. In Fig. 5a, there are persistent hyaloid vessels at P14, after which the aflibercept was injected. The hyaloid vasculature at P18 was barely seen as a consequence of the injection. However, at P21 and P24, a few numbers of vessel segments started to reappear, suggesting a subsequent recovery following a significant decrease in blood flow at P18. The retinal vasculatures showed spatial variations in the vascular development and again showed the retarded development in the direction of the hyaloid vessel persistence at P24. Meanwhile, the choroidal vasculatures did not show any spatial variation. In the fellow eye as shown in Fig. 5b, the hyaloid vasculature gradually regressed over time. The retinal vasculature showed a spatial variation in the vessel density at P14 and the degree of the variation subsequently lessened; densely grown hyaloid vessels and avascular retina are observed particularly in the upper right corner at P14, followed by pronounced hyaloid vessel regression and retinal vascularization in the corresponding direction from P18 to P24. The choroidal vasculature does not show noticeable spatial variation at all time points. The hyaloid vessel volume measurement reveals that the vessel volumes in the injected eyes (red line) decreased rapidly after the injection at P14 as shown in Fig. 5c. Compared to the OIR control eyes (green line), a separately prepared OIR group that has never received the injection in either of the eyes, which showed a significantly reduced vessel volume only at P24, it is noted that the vessel volumes in the fellow eyes (blue line) continuously decreased from P14 to P24 possibly due to the systemic anti-VEGF effect of the aflibercept injection^33,34^. Fig. 5d,g show the correlations between the hyaloid vessel volume and the retinal vessel density. Before the injection at P14, the two groups showed negative correlations as there was no difference between them. In the aflibercept injected eyes, the negative correlation was not observed at P18 and P21 because of the significantly reduced hyaloid vessel volumes, however it is observed again at P24 as the hyaloid vessel segments partly reappeared in the areas of retinal vessel deficiency. In the fellow eyes, the correlation is observed from P18 to P24 to a lesser degree compared to the OIR retina. No correlation was observed between the hyaloid vessel volume and the choroidal vessel density in either group as shown in Fig. 5h–k.

### Retinal and choroidal vascular development over time

**Figure 6:**
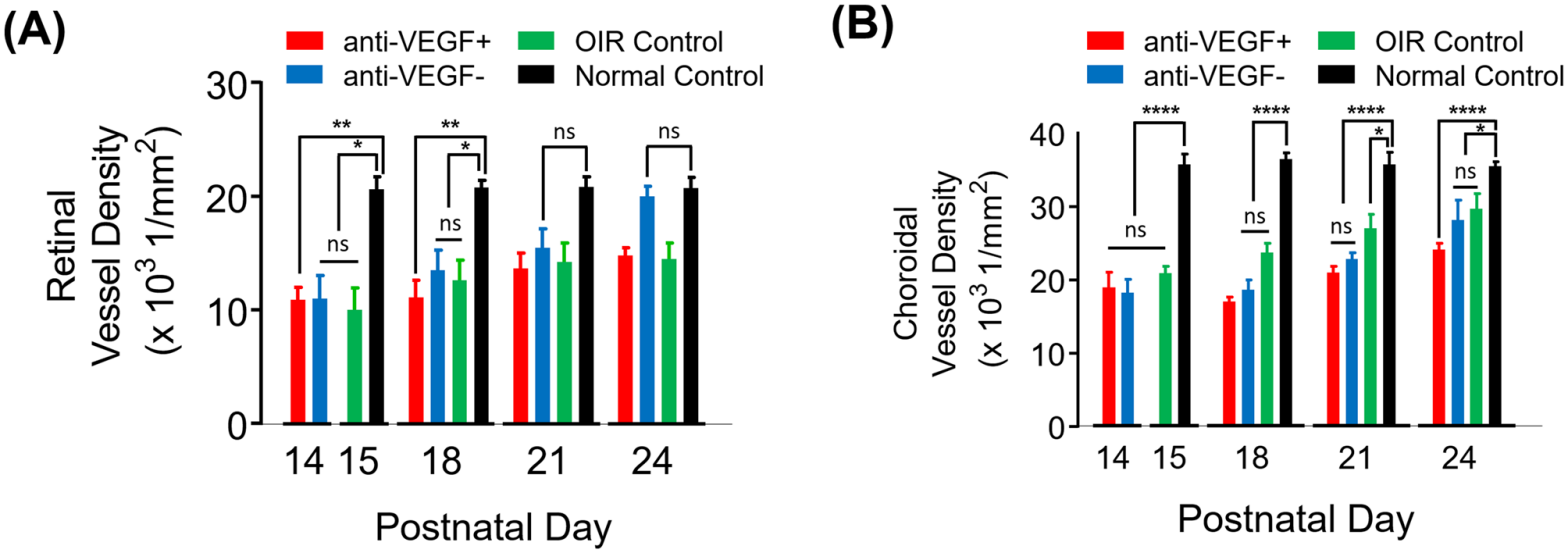
Longitudinal measurements of (**a**) retinal and (**b**) choroidal vessel densities in the normal control, the OIR control without any injection in either of the eyes, the OIR with anti-VEGF injection, and the fellow eyes. *P < 0.05, **P < 0.01, and ****P < 0.0001.

The horizontal distributions of the data points in Figs. 4c–j and 5d–k represent the retinal and choroidal vessel densities, which tend to shift rightward over time except in the normal control group. To further investigate this temporal change, the retinal and choroidal vessel densities in normal and OIR control eyes, which have never received an anti-VEGF injection in either of the eyes, from P15 to P24, and OIR eyes with an anti-VEGF injection and their fellow eyes from P14 to P24 were plotted together in Fig. 6. Compared to the normal eyes at P15, the retinal vessel densities in the anti-VEGF injected eyes and the fellow eyes at P14, and the OIR eyes at P15 were significantly lower because of vasoattenuation in the OIR model^35^ and gradually increased until P24 as shown in Fig. 6a. Despite the effect of the systemically delivered anti-VEGF agent, the retinal vessel density in the fellow eyes increased more rapidly than that in the OIR control eyes. In Fig. 6b, the choroidal vessel density in the OIR control eyes is significantly lower than that in the normal control eyes at P15. It increases monotonically over time but still lower than that in the normal eyes at P24, which is consistent with the previous study^28^. The choroidal vessel densities in the anti-VEGF injected eyes and their fellow eyes were also low at P14 and then increased.

## Discussion

In this study, we performed longitudinal imaging of the rat hyaloid, retinal, and choroidal vasculatures in normal and OIR rat models in vivo using OCTA. The spatiotemporal relationships between the regression of the hyaloid vessels and the development of the retinal and choroidal vessels were investigated for the first time to our knowledge through simultaneous visualization of the hyaloid, retinal, and choroidal vasculatures. Benefited from its ability to perform non-invasive three-dimensional depth-resolved imaging without fluorescent dye injection, OCTA is well suited for investigating the correlations of spatiotemporal vascular changes in different layers.

In the OIR model, a spatial relationship between the proliferation of the hyaloid vessels and the retardation of the retinal vessel development was longitudinally observed. The spatial correlation can be explained that the hyaloid vessels are maintained by the local VEGF expressed from the ischemic immature retina with insufficient blood supply via retinal arteries and capillaries^15^. It is also known that the retinal neuron-derived VEGF maintains hyaloid vasculature via endothelial VEGFR2. It was shown that the genetic depletion of neuronal VEGFR2 resulted in persistent hyaloid vessels in mice via the increased vitreous level of VEGF proteins^14^. Therefore, in this study, strong VEGF production in the less vascularized retinal periphery and weak VEGF production in the well-vascularized retinal periphery might result in the VEGF gradient inside the vitreous cavity leading to asymmetric hyaloid vasculature and its regression. Another evidence is the effect of intravitreal injection of anti-VEGF in OIR eyes. The anti-VEGF injection in OIR eyes showed a pronounced effect on the hyaloid regression in the injected eyes as well as the fellow eyes to a lesser degree. This result is relevant considering the role of VEGF as a vascular survival factor^36^. The depletion of intraocular VEGF caused the regression of the hyaloid vessels and the developing retinal vessels at P18, confirming the VEGF as a key factor of hyaloid vessel maintenance. Considering that the ocular half-life of immunoglobulin G (IgG) to which aflibercept belongs is 0.4 days in rats^37^, the efficacy of anti-VEGF is decreased at P21 and P24 and rebound growth of remnant hyaloid vessels could be observed in the area of the ischemic retina where VEGF is supposed to be produced (Fig. 5a).

The retinal vascularization of the non-injected fellow eyes is also noteworthy. A previous report suggested that the microenvironmental VEGF concentration is a key determinant that induces normal or aberrant angiogenesis^38^. Therefore, it can be considered that the anti-VEGF delivered to the fellow eyes acted in a way that suppresses the overexpression of VEGF in OIR eyes and maintains the physiologic level of VEGF in fellow eyes, facilitating normal retinal vascularization.

There are multiple animal models that mimic the retinal neovascular diseases such as age-related macular degeneration and proliferative diabetic retinopathy^39,40^. However, no animal model completely matches with the human retinal neovascular diseases, and thus, additional in vivo animal models are still necessary for preclinical evaluation of anti-angiogenic agents. In that sense, OCTA-based measurement of hyaloid vessels can be a good in vivo model for the preclinical study of new anti-angiogenic drugs.

It should be clarified that the retinal vessel densities of the OIR control eyes at P21 and P24 were lower than those of the normal eyes as opposed to the result reported by the same group where the vessel densities in the superficial and deep vascular plexus in OIR eyes do not differ from those in normal eyes^28^. It is known that this inter-animal variability on vascular phenotype can be affected by postnatal weight gain and change of nursing behavior of lactating mother due to hyperoxia-dependent stress^32,41^. By carefully controlling intra- and inter- litter variation, one can observe consistent vascular phenotype over multiple measurements.

There are limitations to this study. The imaging field of OCTA is not sufficient to cover the entire vasculatures and it should be noted that the results presented in this study are confined to the central part of the hyaloid, retinal, and choroidal vasculatures. The large hyaloid shadow areas, particularly seen in the early days in the OIR eyes, obscure a significant portion of the retinal and choroidal tissues, making it difficult to observe distinct vascular networks in the OCTA images. Moreover, imaging earlier time points would be beneficial to reflect the various stages of vascular development and regression. Considering that the hyaloid regression and retinal development in rodent eyes complete in the first 2 or 3 weeks after birth, assessing newborn pups is crucial to understand the entire regression process.

In conclusion, OCTA is a promising imaging technique for studying ocular vascular development. Specifically, considering the three-dimensional and temporally changing nature of the hyaloid vasculature, the capability of OCTA for noninvasive and depth-resolved measurement makes it a unique tool over other imaging modalities. Assessing the retinal and choroidal vasculatures in parallel with the hyaloid vasculature is beneficial for an integrated understanding of the intraocular vascular regression and development. The investigation of the anti-VEGF effect in this study further addresses that OCTA can be used to explore various factors playing different roles in the vascular regression and help evaluate the in vivo efficacy of novel ophthalmic anti-angiogenic drugs in future.

## Materials and methods

### Animal protocol

Two groups of Sprague–Dawley (SD) rat pups, room-air raised normal and OIR groups were used in the study. The OIR pups with their nurturing mother were exposed to alternating cycles of 80% oxygen (for 21 h) and 20% oxygen (for 3 h) from P1 to P11 and returned to room-air at P12^42^. The animal protocols of exclusion criteria are described in the previous publication^28^. For each group, OCTA imaging was performed in 5 enrolled eyes at postnatal day 15 (P15), P18, P21, and P24. The animals were anesthetized with intramuscular injection of ketamine (60 mg/kg) and xylazine (3 ~ 4 mg/kg) and the eyes were dilated by topical application of 1% tropicamide. After OCTA imaging at P15, P18, and P21, yohimbine (2 mg/kg) was injected intraperitoneally to prevent corneal calcification which can potentially attenuate OCT signal and degrade image quality^43^. Immediately after OCTA imaging at P24, all animals were euthanized and eyeballs were enucleated for retinal whole mount imaging. To validate the performance of OCTA in visualizing the hyaloid vasculature, fluorescein angiography (FA) imaging was performed in three extra OIR eyes at P24 using a confocal scanning laser ophthalmoscope (CSLO) prototype.

To investigate the effect of anti-VEGF on the hyaloid regression, an OIR group was additionally prepared. An anti-VEGF agent (Aflibercept, 32 µg in 800 nL; Eylea, Bayer Pharma AG, Berlin, Germany) was injected intravitreally into the right eyes at P14 using a nanoliter injector (World Precision Instrument, Sarasota, FL, USA). Both uninjected left eyes and right eyes (n = 5) underwent OCTA imaging at P14 prior to the injection, P18, P21, and P24.

All animal experiments were performed in accordance with the Association for Research in Vision and Ophthalmology statement for the use of animals in ophthalmic and vision research and with approval of institutional animal care and use committee (IACUC) of Korea Advanced Institute of Science and Technology (KAIST) and Seoul National University Bundang Hospital (SNUBH).

### OCTA imaging

**Figure 7:**
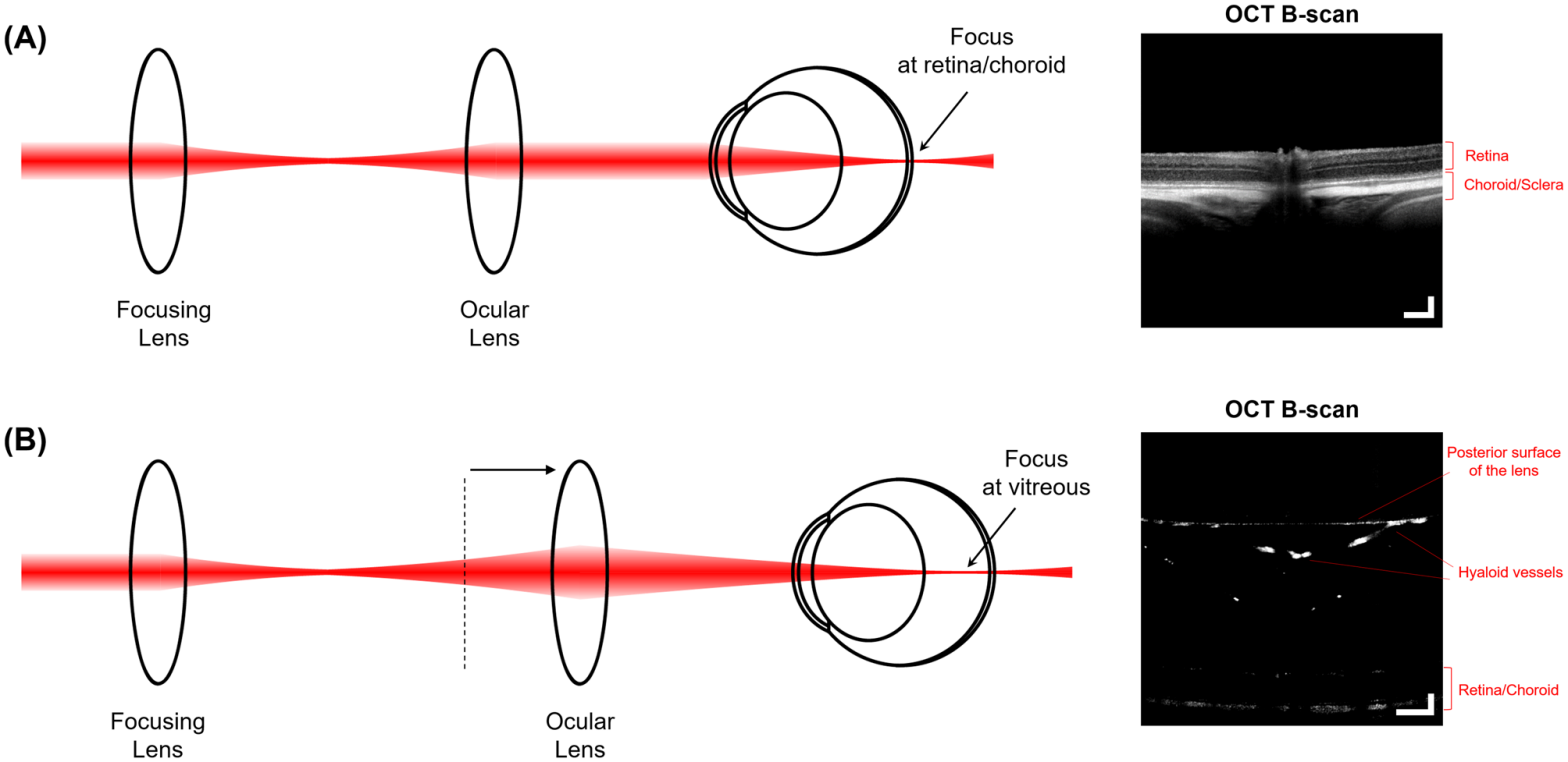
Illustration of the OCT ophthalmoscopes for retinal, choroidal and hyaloid imaging. (**a**) For retinal and choroidal imaging, the ocular lens delivers a collimated beam to the eye, making a focal spot that covers both retinal and choroidal layers. The image on the right is a representative OCT B-scan visualizing the retinal and choroidal layers. (**b**) The ocular lens is translated further away from the focusing lens to deliver a converging beam and makes a focus in the middle of vitreous space. The image on the right is a representative OCT B-scan visualizing the hyaloid vessels in the vitreous space as well as the posterior surface of the lens, retinal and choroidal layers. Scale bars: 200 μm.

A swept-source based OCTA prototype was used in the study. The detailed system specifications are provided in the previous publication^27^. Since the spatial extent of the hyaloid vasculature differs from those of retinal and choroidal vasculatures, two OCTA imaging sessions, each of which was intended to have optimal imaging performance for visualization of hyaloid, and retinal and choroidal vasculatures respectively, were separately and sequentially performed as illustrated in Fig. 7. For retinal and choroidal imaging, the ophthalmoscope produced a collimated beam on the cornea and made a focus on the retinal and choroidal layers (Fig. 7a). For hyaloid imaging, the ocular lens was translated using a manual translation stage (#03-682; Edmund Optics Inc., Barrington, NJ, USA) so that a converging beam on the cornea focused in the middle of the vitreous space (Fig. 7b). The position of the reference mirror was also adjusted accordingly to match the optical path length variation.

Areas of 2 mm × 2 mm for the retinal and choroidal vasculatures and 1.6 mm × 1.6 mm for the hyaloid vasculature centered at optic nerve head (ONH) were scanned. At each of 800 B-scan positions in the slow-axis direction, five B-scans (800 A-lines/B-scan) were repeatedly acquired. After applying a post-processing motion compensation algorithm within each B-scan position^44^, an intensity-variance algorithm was used to calculate decorrelation signals between each pair of consecutive B-scans, generating a total of four OCTA B-scan angiograms^45^. The angiograms were averaged to increase the signal-to-noise of the image. In the hyaloid volumetric angiogram, the posterior surface of the lens and the inner limiting membrane (ILM) were manually segmented using ImageJ software (https://imagej.nih.gov/ij/; provided in the public domain by the National Institutes of Health, Bethesda, MD, USA) and the maximum decorrelation signals between the two segmented boundaries within each A-line were projected to generate a hyaloid OCTA image. In the same manner, in the retinal and choroidal volumetric angiogram, the ILM and the retinal pigment epithelium (RPE) were manually segmented, and the maximum decorrelation signals from the ILM to the RPE and those beneath the RPE within each A-line were projected to generate retinal and choroidal OCTA images, respectively. Since the hyaloid vasculature attenuates OCT beam significantly and leaves local regions of the signal void in the retinal and choroidal layers, these shadow regions were overlaid as blue color in retinal and choroidal OCTA images and excluded from analyses hereafter^28^.

### Quantitative analyses of OCTA image

For hyaloid vessel volume measurement, the decorrelation signals between the posterior surface of the lens and the ILM in each OCTA B-scan angiogram were binarized by applying a median filter with a 5 × 5 kernel followed by an Otsu thresholding in MATLAB software (Matlab; Mathworks, Natick, MA, USA). A total number of voxels with high signals in the 3D binarized angiogram was calculated and converted to vessel volume in a mm^3^ unit (Supplementary Fig. S2a).

To investigate a spatial correlation between hyaloid vasculature and retinal and choroidal vasculatures, all OCTA angiograms were binarized using the Otsu thresholding, after which they were equally divided into eight subsets by four planes intersecting the center of the en face plane (Supplementary Fig. 2b). The hyaloid vessel volume from each subset was measured in the 3D angiogram. The retinal and choroidal vessel densities, which are defined by the ratios of the areas occupied by the blood vessels to the areas occupied by the retinal and choroidal tissues^28^, were calculated in each subset of the OCTA image. The hyaloid vessel volumes for retinal vessel densities and the hyaloid vessel volume for choroidal vessel densities were plotted in 2-dimensional Cartesian coordinate systems to analyze the correlations between the vascular parameters.

### FA imaging

To validate OCTA imaging for hyaloid vasculature, three selected OIR pups that were not enrolled in the longitudinal study underwent FA imaging using a CSLO prototype^27^, immediately after OCTA imaging at P24. Fluorescein sodium (40 mg intraperitoneally; Alcon, Fort Worth, TX, USA) was injected approximately 5 min before imaging. The ocular lens (PlanApoλ, numerical aperture = 0.75; Nikon Corp., Tokyo, Japan) was axially translated to produce a focal spot in the middle of the vitreous and an area of 1.6 mm × 1.6 mm centered at the ONH was scanned.

### Immunofluorescence retinal whole mount imaging

Immediately after OCTA imaging at P24, the eyes were enucleated and fixed in 2% paraformaldehyde. The detailed procedures for tissue preparation and vessel staining are described in the previous publication^28^. After staining, the retinal whole mount was visualized with a confocal microscope (LSM 710; Carl Zeiss, Oberkochen, Germany).

### Statistical analysis

For statistical analysis of hyaloid vessel volume and retinal and choroidal vessel densities, means and standard errors of the mean were calculated. A 2-way ANOVA with Dunnett post-hoc testing was used, and a P-value less than 0.05 was considered statistically significant. For investigating spatial correlations between hyaloid regression and retinal and choroidal vascular developments, Pearson correlation coefficients were calculated between hyaloid vessel volumes and retinal and choroidal vessel densities. All statistical analyses were performed using GraphPad Prism 8 statistical software (GraphPad Prism 8; GraphPad Software, San Diego, CA, USA).

## Supplementary information

Supplementary Information.

Supplementary Video 1.

## References

[1] Ito M, Yoshioka M (1999). Regression of the hyaloid vessels and pupillary membrane of the mouse. Anat. Embryol. (Berl.).

[2] Anand-Apte, B., Hollyfield, J. G. Developmental anatomy of the retinal and choroidal vasculature. *Encyclopedia of the Eye*, 9–15. 10.1016/B978-0-12-374203-2.00169-X (2010).

[3] Lutty GA, McLeod DS (2018). Development of the hyaloid, choroidal and retinal vasculatures in the fetal human eye. Prog. Retin. Eye Res..

[4] Silbert M, Gurwood AS (2000). Persistent hyperplastic primary vitreous. Clin. Eye Vis. Care.

[5] Lang RA, Bishop JM (1993). Macrophages are required for cell death and tissue remodeling in the developing mouse eye. Cell.

[6] Lang R, Lustig M, Francois F, Sellinger M, Plesken H (1994). Apoptosis during macrophage-dependent ocular tissue remodelling. Development.

[7] Mitchell CA, Risau W, Drexler HC (1998). Regression of vessels in the tunica vasculosa lentis is initiated by coordinated endothelial apoptosis: A role for vascular endothelial growth factor as a survival factor for endothelium. Dev. Dyn..

[8] Beebe DC (2008). Maintaining transparency: A review of the developmental physiology and pathophysiology of two avascular tissues. Semin. Cell Dev. Biol..

[9] Kaiser D, Freyberg MA, Friedl P (1997). Lack of hemodynamic forces triggers apoptosis in vascular endothelial cells. Biochem. Biophys. Res. Commun..

[10] Lobov IB (2005). WNT7b mediates macrophage-induced programmed cell death in patterning of the vasculature. Nature.

[11] Kato M (2002). Cbfa1-independent decrease in osteoblast proliferation, osteopenia, and persistent embryonic eye vascularization in mice deficient in Lrp5, a Wnt coreceptor. J. Cell Biol..

[12] Nayak G (2018). Developmental vascular regression is regulated by a Wnt/beta-catenin, MYC and CDKN1A pathway that controls cell proliferation and cell death. Development.

[13] Rao S (2013). A direct and melanopsin-dependent fetal light response regulates mouse eye development. Nature.

[14] Yoshikawa Y (2016). Developmental regression of hyaloid vasculature is triggered by neurons. J. Exp. Med..

[15] Fruttiger M (2007). Development of the retinal vasculature. Angiogenesis.

[16] Saint-Geniez M, D'Amore PA (2004). Development and pathology of the hyaloid, choroidal and retinal vasculature. Int. J. Dev. Biol..

[17] De Schaepdrijver L, Simoens P, Lauwers H, De Geest JP, Charlier G (1989). The hyaloid vascular system of the pig. A light and scanning electron microscopic study. Anat. Embryol. (Berl.).

[18] Strek W (1993). Hyaloid vessels of the human fetal eye. A scanning electron microscopic study of corrosion casts. Arch. Ophthalmol..

[19] Bischoff PM, Wajer SD, Flower RW (1983). Scanning electron microscopic studies of the hyaloid vascular system in newborn mice exposed to O_2_ and CO_2_. Graefes Arch. Clin. Exp. Ophthalmol..

[20] Ritter MR (2005). Three-dimensional in vivo imaging of the mouse intraocular vasculature during development and disease. Investig. Ophthalmol. Vis. Sci..

[21] Ang M (2015). Optical coherence tomography angiography for anterior segment vasculature imaging. Ophthalmology.

[22] Ishibazawa A (2015). Optical coherence tomography angiography in diabetic retinopathy: A prospective pilot study. Am. J. Ophthalmol..

[23] Kashani AH, Lee SY, Moshfeghi A, Durbin MK, Puliafito CA (2015). Optical coherence tomography angiography of retinal venous occlusion. Retina.

[24] Jia Y (2014). Quantitative optical coherence tomography angiography of choroidal neovascularization in age-related macular degeneration. Ophthalmology.

[25] Choi W (2015). Ultrahigh-speed, swept-source optical coherence tomography angiography in nonexudative age-related macular degeneration with geographic atrophy. Ophthalmology.

[26] Liu W (2015). Simultaneous optical coherence tomography angiography and fluorescein angiography in rodents with normal retina and laser-induced choroidal neovascularization. Opt. Lett..

[27] Park JR (2016). Imaging laser-induced choroidal neovascularization in the rodent retina using optical coherence tomography angiography. Investig. Ophthalmol. Vis. Sci..

[28] Kim Y (2018). Oxygen-induced retinopathy and choroidopathy: In vivo longitudinal observation of vascular changes using OCTA. Investig. Ophthalmol. Vis. Sci..

[29] Zhi Z, Cepurna WO, Johnson EC, Morrison JC, Wang RK (2012). Impact of intraocular pressure on changes of blood flow in the retina, choroid, and optic nerve head in rats investigated by optical microangiography. Biomed. Opt. Express.

[30] Soetikno BT (2017). Optical coherence tomography angiography of retinal vascular occlusions produced by imaging-guided laser photocoagulation. Biomed. Opt. Express.

[31] Larrazabal LI, Penn JS (1990). Fluorescein angiography of the newborn rat. Implications in oxygen-induced retinopathy. Investig. Ophthalmol. Vis. Sci..

[32] Stahl A (2010). The mouse retina as an angiogenesis model. Investig. Ophthalmol. Vis. Sci..

[33] Matsuyama K (2011). Effects of intravitreally injected bevacizumab on vascular endothelial growth factor in fellow eyes. J. Ocul. Pharmacol. Ther..

[34] Michalska-Malecka K (2016). Effects of intravitreal ranibizumab on the untreated eye and systemic gene expression profile in age-related macular degeneration. Clin. Interv. Aging.

[35] Penn JS, Tolman BL, Henry MM (1994). Oxygen-induced retinopathy in the rat: Relationship of retinal nonperfusion to subsequent neovascularization. Investig. Ophthalmol. Vis. Sci..

[36] Alon T (1995). Vascular endothelial growth factor acts as a survival factor for newly formed retinal vessels and has implications for retinopathy of prematurity. Nat. Med..

[37] Caruso A (2020). Ocular half-life of intravitreal biologics in humans and other species: Meta-analysis and model-based prediction. Mol. Pharm..

[38] Ozawa CR (2004). Microenvironmental VEGF concentration, not total dose, determines a threshold between normal and aberrant angiogenesis. J. Clin. Investig..

[39] Pennesi ME, Neuringer M, Courtney RJ (2012). Animal models of age related macular degeneration. Mol. Aspects Med..

[40] Liu CH, Wang Z, Sun Y, Chen J (2017). Animal models of ocular angiogenesis: From development to pathologies. FASEB J..

[41] Kim CB, D'Amore PA, Connor KM (2016). Revisiting the mouse model of oxygen-induced retinopathy. Eye Brain.

[42] Deliyanti D (2012). Neovascularization is attenuated with aldosterone synthase inhibition in rats with retinopathy. Hypertension.

[43] Zhou TE (2017). Preventing corneal calcification associated with xylazine for longitudinal optical coherence tomography in young rodents. Investig. Ophthalmol. Vis. Sci..

[44] Guizar-Sicairos M, Thurman ST, Fienup JR (2008). Efficient subpixel image registration algorithms. Opt. Lett..

[45] Lee J, Jiang JY, Wu W, Lesage F, Boas DA (2014). Statistical intensity variation analysis for rapid volumetric imaging of capillary network flux. Biomed. Opt. Express.

