# Supplementary material for: In vivo imaging of the hyaloid vascular regression and retinal and choroidal vascular development in rat eyes using optical coherence tomography angiography
Source: Sci Rep. 2020 Jul 30;10:12901. doi: 10.1038/s41598-020-69765-7 (PMC7393149; doi:10.1038/s41598-020-69765-7)
Supplement: Supplementary file 1 — Supplementary Information. [file 41598_2020_69765_MOESM1_ESM.pdf]

In vivo Imaging of the Hyaloid Vascular Regression and Retinal and Choroidal Vascular Development in Rat Eyes Using Optical Coherence Tomography Angiography

Yongjoo Kim[1,2], Jang Ryul Park[1,2], Hye Kyoung Hong[3], Myounghee Han[4], Jingu Lee[2,5], Pilhan Kim[2,5], Se Joon Woo[3], *Kyu Hyung Park[3], and *Wang-Yuhl Oh[1,2]

[1]Department of Mechanical Enginnereing, Korea Advanced Institute of Science and Technology (KAIST), 291 Daehak-ro, Yuseong-gu, Daejeon 34141, Republic of Korea

[2]KI for Health Science and Technology, Korea Advanced Institute of Science and Technology (KAIST), 291 Daehak-ro, Yuseong-gu, Daejeon 34141, Republic of Korea

[3]Departments of Ophthalmology, Seoul National University College of Medicine, Seoul National University Bundang Hospital (SNUBH), 173-82 Gumi-ro, Bundang-gu, Seongnam, Gyeongi-do 13620, Republic of Korea

[4]Machine Vision R&D Center, Koh Young Technology, Inc., Yongin-si, Gyeonggi-do 16864, Republic of Korea

[5]Graduate School of Nanoscience and Technology, Korea Advanced Institute of Science and Technology (KAIST), 291 Daehak-ro, Yuseong-gu, Daejeon 34141, Republic of Korea

Y. Kim, J.R. Park, and H.K. Hong contributed equally to this work.

W.Y. Oh and K.H. Park are joint corresponding authors.

E-mail: woh1@kaist.ac.kr, jiani4@snu.ac.kr

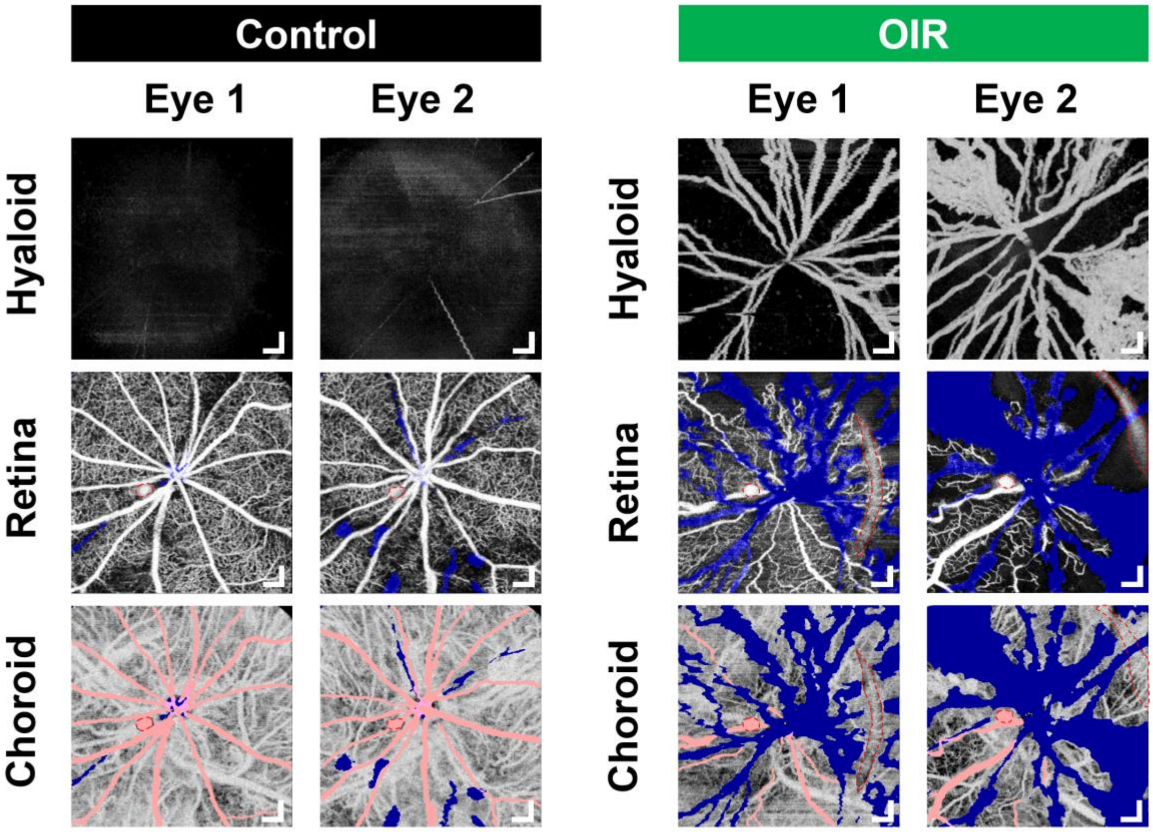

Supplementary Figure 1. Visualization of the hyaloid, retinal, and choroidal vasculatures in the control and OIR eyes at P24. Scale bars: 200 μm.

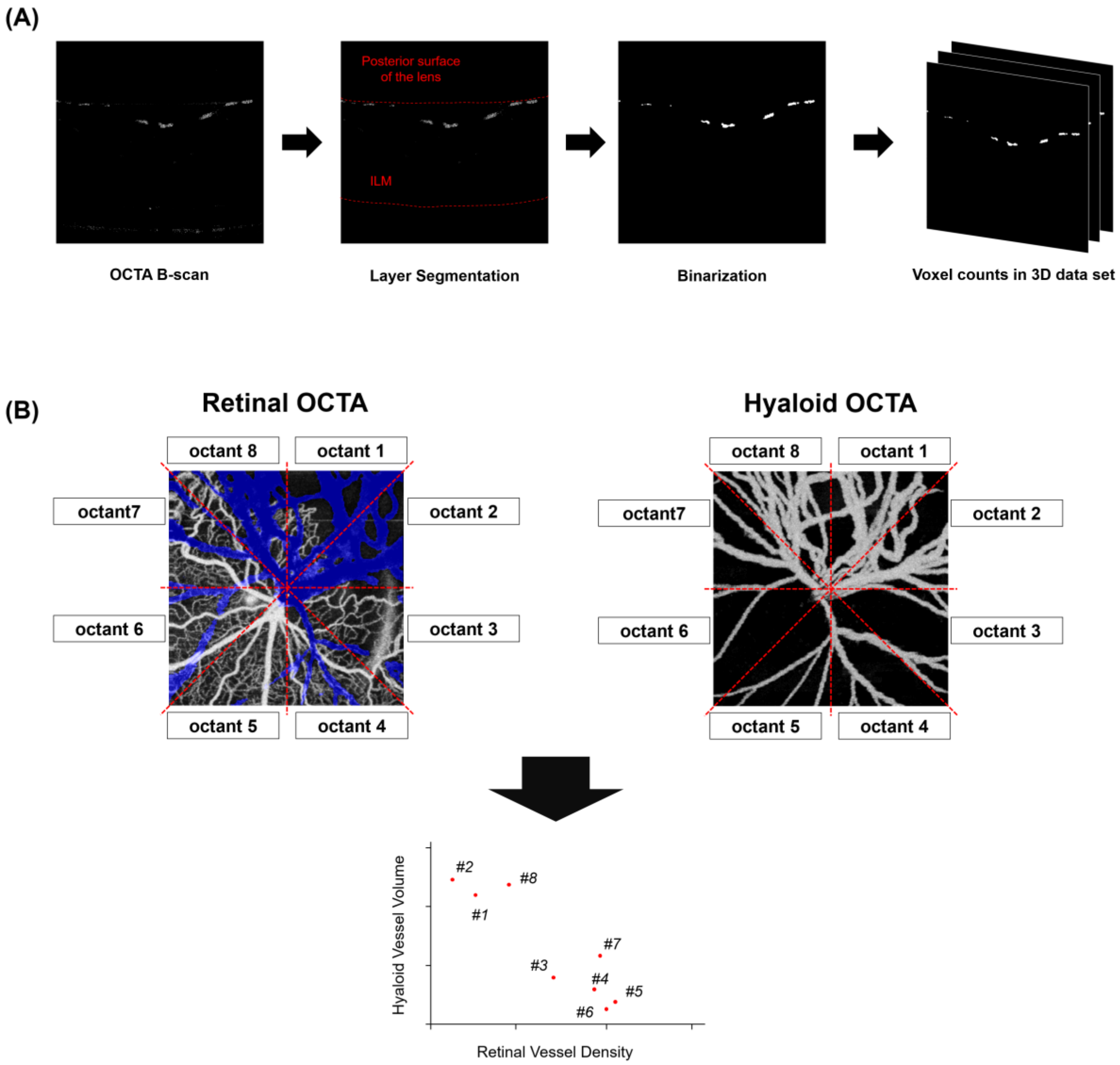

Supplementary Figure 2. Methods for quantitative analysis using OCTA images.
(A) Hyaloid vessel volume measurement. (B) Correlation analysis between the hyaloid vessel volume and the retinal vessel density.

Supplementary Video 1. Provided with additional movie file.
